# SARS-CoV-2 infection and cognition in community-dwelling and nursing home residents in southern Switzerland

**DOI:** 10.1016/j.bbih.2023.100701

**Published:** 2023-11-07

**Authors:** Greta Rizzi, Deborah Pacifico, Serena Sabatini, Anna Maria Annoni, Federico Mele, Sandra Jovic, Luca Piccoli, Laurie Corna, Rebecca Amati, William Pertoldi, Maddalena Fiordelli, Federica Sallusto, Emiliano Albanese

**Affiliations:** a Institute of Public Health, Faculty of Biomedical Sciences, Università Della Svizzera Italiana, Lugano, Switzerland; b Institute of Mental Health, School of Medicine, University of Nottingham, Nottingham, United Kingdom; c Institute for Research in Biomedicine, Università Della Svizzera Italiana, Bellinzona, Switzerland; d Humabs BioMed SA, a Subsidiary of Vir Biotechnology, Bellinzona, Switzerland; e Centre of Competence on Ageing, Department of Business Economics, Health & Social Care, University of Applied Sciences & Arts of Southern Switzerland, Manno, Switzerland; f Istituti Sociali, Chiasso, Switzerland; g Institute of Microbiology, ETH Zurich, Switzerland

**Keywords:** Cognitive COVID, SARS-CoV-2 infection, Cognitive decline, Serological assessment, Neuropsychological assessment, Long COVID

## Abstract

**Background:**

COVID-19 patients can report ‘brain fog’ and may exhibit cognitive symptoms for months after recovery (Cognitive COVID). However, evidence on whether and the extent to which SARS-CoV-2 infection impacts cognition irrespective of COVID-19 course and severity is limited to clinical samples and mainly comes from prognostic studies. We aimed to explore the association between serologically confirmed SARS-CoV-2 infection and cognitive functioning in community-based and institutionalized older adults, irrespective of COVID-19 symptoms.

**Methods:**

We conducted a case-control study nested into two cohorts in Southern Switzerland. Eligible subjects were Italian speaking older adults, without a previous diagnosis of dementia, who underwent serological testing for anti-SARS-CoV-2 antibodies between November 2020 and July 2021. We manually selected age-, sex- and education-matched cases (i.e., individuals with a serologically confirmed SARS-CoV-2 infection), with seronegative controls, and we conducted in-person neuropsychological assessments using validated, highly sensitive cognitive tests.

**Results:**

We completed 38 neuropsychological assessments in a mostly female sample of older adults (Mean age: 83.13 ± 8.95; 86.8% women). 17 were community dwelling individuals while 21 lived in a nursing home. As expected, socio-demographic characteristics of age, gender and educational level were similarly distributed between cases (n = 14) and controls (n = 24). In linear regression models, cases had significantly lower scores in cognitive tasks of memory (β = −0.367, p = 0.023), attention (β = 0.428, p = 0.008) and executive functions (β = 0.326, p = 0.046). We found no significant difference in tests of language and spatial-temporal orientation (all p values > 0.05).

**Conclusions:**

SARS-CoV-2 infection was associated with cognitive impairment in memory, attention, and executive functions in older adults. Our findings are consistent with mechanistic evidence of the neurotropism of the virus and provide empirical support for the “Cognitive COVID” construct also in non-clinical samples. With nearly 800 million COVID-19 cases (in April 2023), and many more infections worldwide, the clinical and public health implications of Cognitive COVID due to SARS-CoV-2 infection may be massive and warrant further epidemiological investigations.

## Highlights

• Cognitive assessment in non-hospitalized COVID-19 cases and seronegative controls.
• SARS-CoV-2 seropositivity in community-dwelling and nursing home older adults.
• Poor memory, attention, and executive functions in seropositive SARS-CoV-2 cases.
• SARS-CoV-2 infection is associated with poor cognition irrespective of symptoms.

## 1 Introduction

COVID-19, the disease caused by SARS-CoV-2 infection, can cause brain fog, or Cognitive COVID which refers to the medium- and long-term sequelae of SARS-CoV-2 infection on cognitive functions including attention, memory, and language (Ritchie and Chan, 2021). Although, the pathophysiological mechanisms of Cognitive COVID are not yet well known, it has been hypothesized that cognitive deficits may be the consequences of an immune dysfunction, including a non-specific neuroinflammation (Spudich and Nath, 2022) or of the neurotropism of SARS-CoV-2, i.e., the ability of the virus to invade and infect the neural tissue (Desai et al., 2022; Hu et al., 2020). Recent estimations suggest that about 45% of people with a SARS-CoV-2 infection may have Long COVID (O'Mahoney et al., 2023), defined as the persistence of symptoms for 12 or more weeks after recovery from COVID-19 (Bellan et al., 2021). Moreover, evidence is rapidly expanding about the long-term neurological (headache, smell or taste disorders) (Chen et al., 2021; Nalleballe et al., 2020), neuropsychiatric, and psychological (Mazza et al., 2021; Nalleballe et al., 2020; Vindegaard and Benros, 2020) complications of SARS-CoV-2 virus. However, the mid-to long-term consequences on cognition have received comparatively less attention. Limited evidence available suggests that COVID-19 may impair several cognitive abilities, particularly memory, attention and executive functions (Alemanno et al., 2021a; Almeria et al., 2020; Chaumont et al., 2020; Helms et al., 2020; Premraj et al., 2022; Reford et al., 2022). Evidence is not only sparse but also somewhat inconsistent (Zhou et al., 2021). Although decreased verbal fluency (Beaud et al., 2021) and reduced processing speed may be part of Cognitive COVID (Almeria et al., 2020; Iodice et al., 2021), memory deficits, attentional disorders, and mental fog are the most commonly self-reported symptoms (Davis et al., 2021a; Misra et al., 2021; Premraj et al., 2022). Cognitive COVID is very common. Estimations based on both objective cognitive assessments (Almeria et al., 2020) and self-reported cognitive difficulties (Davis et al., 2021a; Liguori et al., 2020) suggest that Cognitive COVID may be experienced by up to a third of COVID-19 patients after recovery, but current prevalence estimates vary considerably between 40% (Reford et al., 2022) and 88% in the Long COVID population (Davis et al., 2021a). Cognitive difficulties may endure several months (Almeria et al., 2020; Ferrucci et al., 2021), interfere with the ability of individuals to conduct daily activities (Reford et al., 2022), and lead to increased use of healthcare facilities in terms of increased help-seeking behavior to obtain pharmacological treatments for symptoms (McNaughton et al., 2022). Therefore, Cognitive COVID likely exacerbates the societal and economic impact of the COVID-19 pandemic irrespective of the dynamics and spreading of infections.

Cognitive COVID is important but difficult to investigate both from a clinical and public health perspective. As said, current evidence on Cognitive COVID remains limited, and it is mainly based on data collected in hospitalized patients in the post-acute phase of the infection (Almeria et al., 2020; Ferrucci et al., 2021; Mazza et al., 2021), despite only a minority of COVID-19 patients are hospitalized. This clinical perspective may be biased, and we still know very little on the consequences of SARS-CoV-2 on cognition in asymptomatic or mild non-hospitalized cases. Comprehensive monitoring of cognitive deficits should be extended to infected individuals irrespective of COVID-19 symptoms. Measurement bias may not be excluded either because existing studies mainly relied on participants’ performance on general neuropsychological screening tests such as the Mini Mental State Examination (MMSE) or the Montreal Cognitive Assessment (MoCA) (Alemanno et al., 2021a; Patel et al., 2021) although these may prove insensitive to subtle changes in cognition, particularly if impairment is mild and selective (Beaud et al., 2021; Lynch et al., 2022). Furthermore, older adults are often underrepresented in studies evaluating the potential consequences of COVID-19 on cognition because of a supposed difficulty in disentangling age-related cognitive changes or impairments from cognitive difficulties due to COVID-19 (Prendki et al., 2020). Finally, evidence in older adults who live in long-term care facilities, in whom both cognitive impairment and SARS-CoV-2 infections are very common, is non-existent.

Our aim was to study the association of SARS-CoV-2 infection with cognitive sequelae, overcoming the above listed limitations and reducing proneness to biases of previous studies. We conducted a case-control study at the community level and in a nursing home for older adults. We hypothesized that there is an association between serologically confirmed infections and cognitive impairment, irrespective of COVID-19 symptoms and severity. In order to provide empirical evidence to better define the Cognitive COVID construct, we also sought to describe the cognitive profile of people over the age of 65 previously affected by COVID-19.

## 2 Methods

### 2.1 Study design and setting

We conducted a nested case-control study within the Corona Immunitas Ticino and Cov-risk nursing homes cohort studies in Southern, Italian-speaking Switzerland (i.e., Ticino canton). Both studies have been previously described (West et al., 2020) and entail repeated serological ELISA tests of anti-SARS-CoV-2 antibodies, and an array of COVID-19 related measures, including exposure to infection, symptoms, and impact.

Between July 2021 and January 2022, we drew cases (i.e., people with a serologically confirmed infection, irrespective of symptoms) and manually matched them with controls (i.e., people without SARS-CoV-2 antibodies due to infection, in their serum) and conducted in depth, in-person neuropsychological assessments. Interviews lasted 45 min on average and were all conducted by a purposely trained junior psychologist (GR).

The study was approved by the Ticino Cantonal Ethics Committee (Project ID, 2021-00742) and found to comply with the principles of research involving human people.

### 2.2 Study population

Participants were Italian speaking older adults (≥65 years) without a previous diagnosis of dementia, who underwent serological testing between November 2020 and July 2021.

We selected cases (seropositives) in the source populations using a *Simple Random Sampling* technique*.* We manually matched seronegative controls with cases based on age, gender, and years of education, with a planned one-to-one control to case ratio. We included both community-dwelling older adults and residents living in a nursing home. The planned sample size was 35 participants, based on a priori power analysis implemented in Statulator (2014) (http://statulator.com/SampleSize/ss2M.html) using the following parameters: probability level (α) 0.05, and statistical power (1 - β) 0.80. We sampled and contacted 50 people, and 38 agreed to take part in the study.

All participants (or a legal representative) provided handwritten signing of the informed consent document before the interviews.

### 2.3 Variables and measures

We collected socio-demographic characteristics (age, date of birth, gender, and educational level), and information related to COVID-19 plausible symptoms (fever, cough, sore throat, loss of taste and/or smell etc.) and severity of disease with online self-reported questionnaires. Healthcare staff could assist older adults in data collection based on need. For residents living in a nursing home a staff member completed the questionnaires on their behalf. For the cognitive assessment schedule, we assembled a battery of cognitive tests related to the Cognitive COVID construct (Ritchie and Chan, 2021). We used previously validated and highly sensitive neuropsychological tests to comprehensively assess cognitive functioning. The battery assessed the following cognitive domains: (a) memory, with the CERAD 10 words list learning test (Sosa et al., 2009); (b) attention, with Trail Making test part A and B (TMT) (Siciliano et al., 2019), (c) executive functions with the shortened version of the Stroop test (Caffarra et al., 2002), (d) language, with semantic and phonemic verbal fluency tasks (e.g. Number of words in 1 min) (Costa et al., 2014; Sosa et al., 2009); (e) visual-spatial perception through the Clock drawing test (Shulman, 2000), and bespoken standard questions about spatial-temporal orientation. We favored instruments with little ceiling effects, and minimally age confounded. We accounted for age and years of education in test execution using normative data and standardized the cognitive test scores accordingly. We also administered the EURO-D scale from the Geriatric Mental State exam (Copeland et al., 2002) to enquire about participants’ mental health status. The EURO-D is a validated, 12-item scale (Castro-Costa et al., 2008) for the evaluation of depression in later life.

To minimize bias, the interviewer was blinded by design about grouping of participants (serologically confirmed SARS-CoV-2 infection).

### 2.4 Statistical analysis

We used IBM® SPSS Statistics version 25 (IBM Corp. Released, 2019. IBM SPSS Statistics for Windows, Version 26.0. Armonk, NY: IBM Corp.) for all statistical analysis and set statistical significance at 0.05.

We computed summary statistics for normally distributed continuous variables as means ± standard deviation (SD), or medians with min/max ranges in case of a skewed distribution and conducted between groups comparisons using Student's *T*-test for independent samples, or the Mann–Whitney non-parametric test, based on departure from normality.

Next, we ran separate linear regressions models to investigate the relationships between seropositivity (independent variable) and cognitive test performance (continuously distributed dependent variables of cognitive tests).

TMT and Stroop test outcomes were considered as binary variables (normal vs impaired score), based on their respective normative grids (Caffarra et al., 2002; Siciliano et al., 2019). For binary outcome measures we computed Odd Ratios (ORs) to compare the frequency of the cognitive outcome in subjects with and without a previous SARS-CoV-2 infection.

### 2.5 Data availability

Anonymized data, including raw and analyzed data, and materials not published within this article will be made available by request from any qualified investigator for *bona fide* uses.

## 3 Results

### 3.1 Study sample: descriptive statistics

A total of 38 older adults took part in the study between July 2021 and January 2022; 21 were nursing homes’ residents, 17 lived in the community. Mean age was 83.13 (SD: ± 8.95), ranging between 68 and 99 years. Most of the study participants were women (86.8%). The characteristics of the actual sample cases (n = 14) and matched controls (n = 24) are summarized in Table 1. By design, cases and controls had similar socio-demographic characteristics of age, gender, and years of education and similar EURO-D total scale score distributions for depression assessment (all p values > 0.05). However, our actual sample was significantly differently distributed according to the place of residence (p value = 0.004) and most cases lived in a nursing home (85.7%). Cases were all in the range from asymptomatic to moderate symptoms and did not require hospitalization for treatment of COVID-19.

**Table 1:** Sociodemographic characteristics of cases and controls.

		**SARS-CoV-2 infection**		**Statistics**
		**Cases** (n = 14)	**Controls** (n = 24)	**p-value**^a^
Age, mean ± SD		88.21 **±** 5.09	80.17 **±** 9.44	0.309
				
Gender, % (n)	Male Female	14.3% (2) 85.7% (12)	12.5% (3) 87.5% (21)	0.875
				
Years of education, % (n)	1–5 years 6–8 years 9–13 years >13 years	28.6% (4) 28.6% (4) 35.7% (5) 7.1% (1)	12.5% (3) 45.8% (11) 37.5% (9) 4.2% (1)	0.559
				
EURO-D – Depression, mean ± SD		4.42 **±** 2.91	3.13 **±** 2.53	0.089
				
Place of residence, % (n)	Nursing home	85.7% (12)	37.5% (9)	0.004
	Community-dwelling	14.3% (2)	62.5% (15)	

a A Student's *T*-test for independent samples or a Chi-square test was run according to the variables.

### 3.2 Neuropsychological findings

We completed the full neuropsychological battery with all participants, except for one of them who could not complete the Trail Making Test Part A, and 10 participants who could not finish the Trail Making Test Part B. Because participants were not able to perform the tests, we coded missing values as impaired scores. In linear regression models, cases obtained considerably lower scores in attention (β = 0.428, p = 0.008), executive functions (β = 0.326, p = 0.046), and memory tasks (β = − 0.367, p = 0.023). We found no significant differences between cases and controls in language and orientation tasks. Detailed neuropsychological test battery results, adjusted by age and years of education, are reported as mean ± SD in Table 2. Then, we calculated Odd Ratios for impaired execution of the Trail Making Test and Stroop Test, respectively using normative cut-offs of normal and impaired performance. Based on the Italian normative cut-off (Siciliano et al., 2019), at the Trail Making Test, 64.3% of cases obtained an impaired score compared to 17.39% in controls. Similarly, at the Stroop test (Caffarra et al., 2002), 64.3% of cases obtained a score below the threshold; the percentage dropped to 20.8% in controls. After adjustment for age and education, SARS-CoV-2 infection was associated with an increased likelihood of an impaired test performance both in the Trail Making Test (OR: 6.84, 95% CI: 1.57–29.80) and the Stroop test (OR: 2.52, 95% CI: 0.65–9.83).

**Table 2:** Cognitive outcomes in cases and controls (reported as mean ± SD) by cognitive domain and neuropsychological tests^a^.

**Cognitive domains (Test)**	**SARS-CoV-2 infection**		**Statistics**					
	**Cases**	**Controls**	**Beta**	**R**^**2**^	**F**	**p value**	**95% CI**	
							**Lower**	**Upper**
**Memory** (10-words list learning test)								
- Immediate recall - Delayed recall - Total Memory Index	9.50 (6.89) 2.14 (2.54) 11.64 (9.06)	14.25 (5.51) 3.88 (2.51) 18.25 (7.83)	−0.363 −0.323 −0.367	0.132 0.104 0.135	5.457 4.183 5.613	0.025 0.048 0.023	−8.87 −3.45 −12.26	−0.63 −0.01 −0.95
**Attention** (Trail Making test)								
- Part A - Part B - B-A	211.50 (252.08) 181.00 (186.75) 23.00 (130.35)	52.08 (85.12) 78.90 (117.49) 48.42 (85.15)	0.428 0.318 −0.116	0.183 0.101 0.014	7.854 2.931 0.357	0.008 0.099 0.555	43.94 −20.48 −112.86	274.89 224.67 62.01
**Executive functions** (Stroop test)								
- Interference of time	88.18 (48.36)	56.80 (42.90)	0.326	0.106	4.274	0.046	0.60	62.18
**Language** (fluency)								
- Semantic fluency - Phonemic fluency	11.38 (6.41) 9.71 (5.55)	15.46 (7.63) 13.13 (4.87)	−0.266 −0.315	0.071 0.099	2.672 3.849	0.111 0.058	−9.13 −6.95	0.99 0.12
**Orientation**								
- Spatial orientation - Temporal orientation	1.79 (0.58) 2.36 (1.55)	1.87 (0.45) 3.04 (1.37)	−0.088 −0.230	0.008 0.053	0.283 2.011	0.598 0.165	−0.43 −1.66	0.25 0.29

a All tests were age-, sex- and education-standardized, based on normative data.

Age did not significantly modify the association of seropositivity with cognitive functions, for all cognitive tests.

## 4 Discussion

Our observations seem to confirm that SARS-CoV-2 infection may lead to long-term sequalae and affect cognition. The cognitive effects of SARS-CoV-2 are common even if it is still not clear how cognitive impairment evolves after recovery from infection. In this nested case-control study, we assessed the association between serologically confirmed SARS-CoV-2 infection and cognitive functions in an older adults' sample. The study aimed at exploring if and which cognitive domains were affected by the virus up to several months following the infection and irrespective of COVID-19 symptoms in both community-dwelling and nursing homes’ residents older adults. The neurotropism of SARS-CoV-2 provides a solid mechanistic presumption for our research hypotheses.

In a mostly female sample of asymptomatic to moderate COVID-19 older adults’ cases and matched seronegative controls, we found that previously infected individuals exhibited cognitive impairment entailing fewer words remembered in memory tasks and more time required to complete attention and executive functions tasks. Lower cognitive performance in cases was up to 4-fold compared to what we observed in controls, independent from the age of participants. The present findings are consistent with previous demonstration of the neurotropism of SARS-CoV-2 (Stein et al., 2022; Sun et al., 2020), which can invade and affect the central nervous system (Desai et al., 2022; Poyiadji et al., 2020), causing long-term sequelae, including cognitive impairment (Desai et al., 2022). Our observations on the most affected cognitive domains (i.e. memory, attention and executive functions) are in line with what has been found in different COVID-19 patients (Alemanno et al., 2021a; Almeria et al., 2020; Helms et al., 2020; Mazza et al., 2021; Pinna et al., 2020). However, a medium-long term impact of the virus more widely distributed on cognition cannot be excluded, including deficits in information processing speed and language, as already reported by other studies (Almeria et al., 2020; Beaud et al., 2021; Iodice et al., 2021). Even if neuropsychological testing across cognitive domains is taxonomic in nature, it may not reflect the complexity of brain-behavior relationships. For example, the Trail Making Test - often used to evaluate attentional skills - involves other cognitive functions such as visuospatial processing, coordination, and graphomotor speed (Bowie and Harvey, 2006). Furthermore, whether infection is associated with difficulties in different cognitive domains has not yet been established and requires further investigation.

Comparisons with other studies are not straightforward because of the methodological heterogeneity. After the first pandemic wave, literature was largely dominated by case reports and small case series conducted in COVID-19 hospitalized patients with moderate to severe symptoms (Almeria et al., 2020; Chaumont et al., 2020; Mazza et al., 2021; Woo et al., 2020). We focused on SARS-CoV-2 infection not on COVID-19, and our cases were mainly a- or pauci-symptomatic older adults. Our findings suggest that Cognitive COVID can occur in previously infected individuals irrespective of the course and severity of COVID-19. Null associations between SARC-CoV-2 infection and cognitive impairment have been previously reported but may be spuriously due to the young age of the study samples (between 30 and 64 years) (Mattioli et al., 2021; Zhou et al., 2021). While the occurrence of Cognitive COVID in infected individuals may increase with age, it is also possible that subtle cognitive impairments are more difficult to detect at younger ages (‘floor effect’, opposite of the ceiling effect, may not be excluded). Nevertheless, there is ample evidence that older adults are more likely to suffer not only from a more severe course of COVID-19 and more severe respiratory symptoms but also delirium (Batty et al., 2020; Misra et al., 2021), which may precede or co-occur with dementia, and impact on cognitive test results. Whether and the extent to which Cognitive COVID and dementia share similar underpinning structural and functional brain damages is worth investigating, not least because the former might exacerbate the latter. Furthermore, our results suggest that older adults who contracted the SARS-CoV-2 virus may be more vulnerable to cognitive decline, irrespective of COVID-19 symptoms and up to several months after recovery from the disease. Should SARS-CoV-2 infection increase dementia risk, the public health implications would be enormous, because billions of people have been infected worldwide.

Epidemiological research on Cognitive COVID is in its infancy, but inconsistent results are likely attributable to measurement. Most of previous studies assessed cognitive deficits following COVID-19 based on self-reporting of both infection and perceived cognitive functioning (Ceban et al., 2022; Crivelli et al., 2022; Premraj et al., 2022) or used generic screening cognitive tests (Mattioli et al., 2021; Patel et al., 2021). Information and measurement bias are likely. We sought to overcome these biases in our study. We established caseness, that is previous infection, based on a valid and reliable serological test, and conducted in-person cognitive assessments using objective and validated neuropsychological measures chosen based on their reliability and sensitivity.

Our study has some worth noting limitations. First, the small sample size of our study may have hindered our ability to detect all true virus-cognition associations. Even if we used a multi-stage approach to contact and attempt to recruit one control per case from the source population, response rate was not high enough to maintain the anticipated ratio and matching of cases and controls in the study sample. Based on the limited number of cases, we selected more controls per case ended up with an almost 1:2 ratio, in order to maximizing the statistical power of the study (Setia, 2016). This is a case control study, with limited external validity by design. Our findings are somewhat preliminary and should be generalized with caution and only to similar populations.

Second, baseline measures of the study participants' cognitive functioning were not available. Issues of directionality may exist that limit any causal speculations for the association between SARS-CoV-2 infection and cognitive deficits. Nevertheless, reverse causality, that is cognitive impairment leading to infection, seems unlikely in our sample because we excluded people with dementia by design. We acknowledge that whether those with poorer cognition were at greater risk of infection because of general poor health may be difficult to disentangle, and evidence is lacking on the association of poor health with adherence to infection preventive measures. Furthermore, although the age difference between cases and controls was not significant and tests were all age-standardized, it cannot be ruled out that age played a role in subtle differences in neuropsychological performance.

Third, most of our cases were nursing home residents, reflecting greater spread and circulation of infection in long-term residential settings compared to communities (Amati et al., 2023; Corna et al., 2022). However, place of residence may have had an impact on cognitive performance and the cognitive decline could be a consequence of social isolation and the strict confinement measures adopted to prevent contagion (Pérez-Rodríguez et al., 2021).

Again, we did not have and could not adjust our statistical models for dysmetabolic measures (including high Body Mass Index, an indicator of global adiposity) or comorbid conditions (such as cardiovascular diseases, diabetes, and hypertension) that may be associated with poorer cognition and cognitive decline (Dye et al., 2017; Livingston et al., 2020). This is a limitation that we share with previous studies (Almeria et al., 2020) which is though unlikely to contribute significantly to residual confounding. While poor health in older adults is associated with more severe courses of COVID-19, evidence about risk of SARS-CoV-2 infection remains highly erratic (Vulturar et al., 2022).

We found that non-hospitalized, SARS-CoV-2 positive older adults had worse cognitive performance compared to immunonaive counterparts, across a variety of cognitive domains, including memory, attention and executive functioning, and visuospatial processing speed. Collectively, our findings extend previous observations in hospitalized COVID-19 patients and relate the cognitive sequalae of the disease to SARS-CoV-2 infection irrespective of symptoms. This provides empirical support for the “Cognitive COVID” construct, and it is consistent with mechanistic evidence of the potentially detrimental neurotropism of SARS-CoV-2 (Zhou et al., 2021). Our results have potential clinical and public health implications. As mentioned earlier, Cognitive COVID might contribute to dementia risk in populations, and may even reduce the buffering potential of cognitive reserve in individuals, lowering the latency between brain damage and disease onset in cognitively healthy individuals or with mild cognitive impairment, a transitioning state between normal cognitive function and dementia. Next, Cognitive COVID may accompany Long COVID, and have remarkable influence on help-seeking behaviors, treatment adherence, and quality of care (O'Mahoney et al., 2023). Hence, the number of people accessing specialized health services is expected to increase and become a noteworthy health challenge after the pandemic. Further research is needed to explore the cost-utility of screening for cognitive deficits in those who exhibit Long COVID symptoms or signs, and potentially in all those who got infected.

Research on Cognitive COVID is in its infancy. We need basic sciences and clinical studies to advance knowledge on mechanisms, etiology, and timing of the phenomenon. The role of medical, psychological, and social/environmental variables that may modulate the risk and expression of Cognitive COVID deserves further investigation. Moreover, longitudinal studies may combine serological testing and objective cognitive assessments with neuroimaging techniques to explore brain structural damage. On the other hand, epidemiological evidence is needed to estimate the prevalence of Cognitive COVID in previously infected individuals, and study potential risk and protective factors.

## 5 Conclusions

Patients with COVID-19 may experience cognitive difficulties (Cognitive COVID) as they recover from the disease. Existing evidence comes mainly from clinical studies. Our data describe an association between serologically confirmed SARS-CoV-2 infection and cognitive impairment, in memory, attention, and executive functions in older adults. It provides empirical knowledge on the Cognitive COVID construct in a non-clinical sample of older adults, supporting the relationship between poor cognition and infection, irrespective of the severity of symptoms. With nearly 800 million COVID-19 cases, Cognitive COVID societal impact may be massive. We need more longitudinal data on the distribution, frequency, and impact of the cognitive post-infection manifestations, as well as on their determinants and modulators to address epidemiological and mechanistic gaps. This knowledge can inform future studies, and surveillance strategies for Cognitive COVID, as well as help in clinical practice in terms of timely diagnosis and treatment options.

## Funding

This research did not receive any specific grant from funding agencies in the public, commercial, or not-for-profit sectors.

## Declaration of competing interest

The authors have no declaration of competing interest.

## Data Availability

Data will be made available on request.
